# Optical-Fiber Power Meter Comparison Between NIST and PTB

**DOI:** 10.6028/jres.108.033

**Published:** 2003-10-01

**Authors:** I. Vayshenker, H. Haars, X. Li, J. H. Lehman, D. J. Livigni

**Affiliations:** National Institute of Standards and Technology, Boulder, CO 80305 USA; Applied Radiometry Section, Physikalisch-Technische Bundesanstalt, Braunschweig, Germany; National Institute of Standards and Technology, Boulder, CO 80305 USA

**Keywords:** calibration, cryogenic radiometer, fiber, international comparison, optical fiber, optical power meter, uncertainty

## Abstract

We describe the results of a comparison of reference standards between the National Institute of Standards and Technology (NIST-USA) and Physikalisch-Technische Bundesanstalt (PTB-Germany) at nominal wavelengths of 1300 nm and 1550 nm using an optical-fiber cable. Both laboratories used thermal detectors as reference standards. A novel temperature-controlled, optical-trap detector was used as a transfer standard to compare two reference standards. Measurement results showed differences of less than 1.5 × 10^−3^, which is within the combined uncertainty for both laboratories.

## 1. Introduction

In a previous paper [1], we reported results of a comparison between NIST and PTB of reference standards used in the calibration of optical-fiber power meters. That comparison was performed with collimated laser beams at 1302 nm and 1546 nm, and did not address additional considerations that arise when a divergent beam, such as that exiting an optical fiber, is used. Here we address that additional issue with a further comparison.

For optical-fiber power meter measurements, the primary standards of both NIST and PTB are cryogenic radiometers that have uncertainties of about 10^−4^. Partly because these primary standards cannot be used with divergent beams, both laboratories use thermal detectors as reference standards in providing calibration services. These reference standards are calibrated against the cryogenic radiometers using collimated beams, but are used with divergent beams.

In the study reported here, the reference standards maintained by our two laboratories were compared using beams from an optical fiber and germanium photodiodes mounted in a trap structure that has been shown to provide a uniform response over a wide field of view [2]. The Ge-trap detector was calibrated first at NIST against the NIST reference standard, then at PTB against the PTB reference standard, and then again at NIST. The same lasers, operating at 1302 nm and 1546 nm, and optical-fiber cable were used at both sites. Both laboratories employed a substitution method for their measurements.

## 2. Transfer Standard

**Fig. 1:**
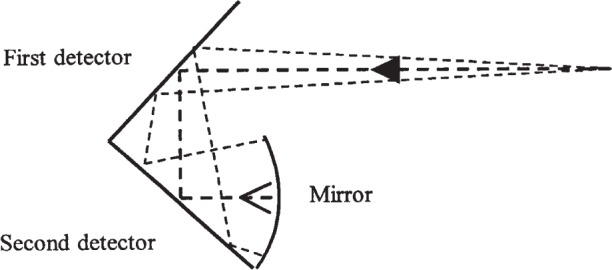
Germanium-trap detector.

For this comparison we used a transfer standard designed and built by NIST. The transfer standard, depicted in Fig. 1 is an optical-trap detector consisting of two germanium photodiodes and a spherical mirror. The trap detector has two, 10 mm diameter, Ge photodiodes and a 15 mm diameter, concave mirror (40 mm focal length) of aluminum coated with magnesium fluoride.

The photodiodes are oriented relative to the entrance aperture so that the principal ray of incoming radiation strikes each diode once at a 45° angle of incidence and then reflects from the concave mirror back again onto the photodiodes in reverse order. The photodiodes and mirror are contained in a thermally stable package.

## 3. NIST Measurement System

**Fig. 2:**
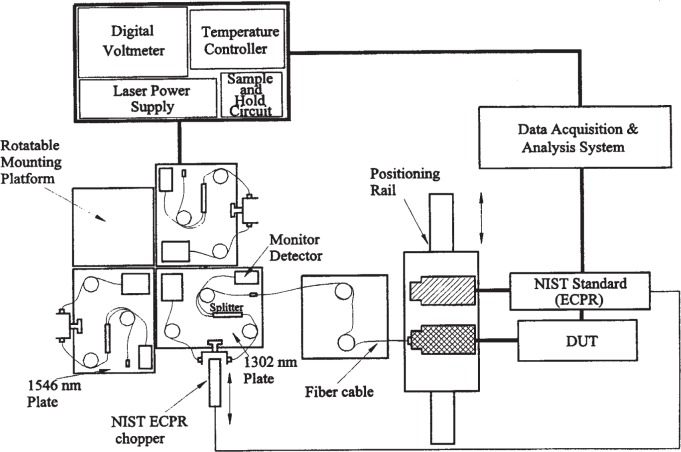
NIST measurement system.

The NIST measurement system, described in [3] and depicted in Fig. 2 consists of fiber-pigtailed laser sources at wavelengths of 1302 nm and 1546 nm, a reference optical-fiber cable, and a positioning stage for comparing the NIST reference and transfer standards. The output of each laser source is transmitted through a fiber to a fiber splitter from which about 1 % of the power travels through a fiber to a monitor detector. The remaining 99 % of the power is transmitted through another fiber to the reference optical-fiber cable.

The NIST reference standard [4] is an electrically calibrated pyroelectric radiometer (ECPR), which had been previously calibrated against a primary standard, the NIST Laser Optimized Cryogenic Radiometer (LOCR). The ECPR is a thermal detector that has an absorbing coating that causes the ECPR to be spectrally insensitive over the wavelength region of 1300 nm to 1550 nm.

## 4. PTB Measurement System

**Fig. 3:**
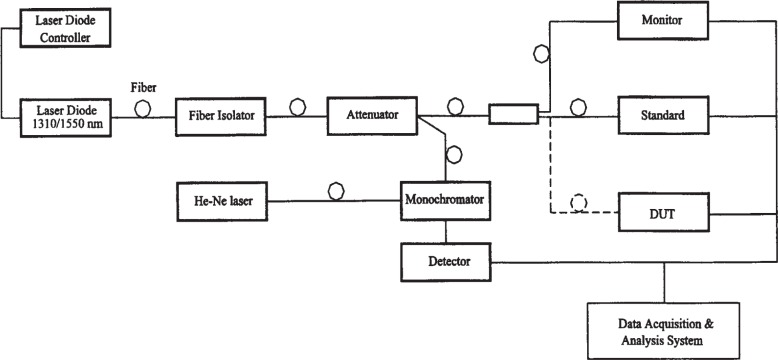
PTB measurement system.

The PTB measurement system depicted in Fig. 3 is similar to the NIST system. It consists of fiber-pigtailed laser sources at wavelengths of 1302 nm and 1546 nm, a reference optical-fiber cable, and a positioning stage for comparing the PTB reference and transfer standards. A fiber splitter and a monitor detector are used to monitor the power during the calibrations. PTB reference and transfer standards are placed together on a computer-controlled positioning stage.

The PTB reference standard described in [5] is a thermopile-based detector that has been calibrated against a silicon-trap detector, which had been previously calibrated against the PTB cryogenic radiometer.

## 5. Results of the Comparison

The NIST and PTB reference standards were compared using the germanium-trap transfer standard, described earlier, and a reference optical-fiber cable at wavelengths of 1302 nm and 1546 nm. The power was approximately 100 µW or −10 dBm. At NIST, six measurement runs were taken both at a wavelength of 1302 nm (relative standard deviation of 0.8 × 10^−3^) and at a wavelength of 1546 nm (relative standard deviation of 0.7 × 10^−3^). At PTB, five measurement runs were taken both at a wavelength of 1302 nm (relative standard deviation of 0.7 × 10^−3^) and at a wavelength of 1546 nm (relative standard deviation 0.3 × 10^−3^). The results of the comparison are given in Table 1.

The standard uncertainties for the PTB optical power measurements were evaluated in accordance with [6] and the standard uncertainties of the NIST measurements were evaluated in accordance with [7]. At 1302 nm the difference between the NIST and PTB results was 2 × 10^−4^, and at 1546 nm the difference was 1.3 × 10^−3^. The NIST combined standard uncertainty was 1.1 × 10^−3^ at 1302 nm and 1.8 × 10^−3^ at 1546 nm, while that of PTB was 1 × 10^−3^ at 1302 nm and 1.5 × 10^−3^ at 1546 nm. The observed interlaboratory differences are less than the stated combined standard uncertainties for both laboratories.

## Figures and Tables

**Table 1:** Results of NIST and PTB comparison

Source wavelength/nm	100 × Relative difference	100 × NIST rel. combined standard uncertainty	100 × PTB rel. combined standard uncertainty
1302	0.02	0.11	0.10
1546	−0.13	0.18	0.14
